# Positive mindset and exercise capacity in school-aged children and adolescents with congenital heart disease

**DOI:** 10.3389/fped.2023.1133255

**Published:** 2023-06-02

**Authors:** Tracy Curran, Rachel Losi, Jennifer Pymm, Julie Ann O’Neill, Nicole Goveia, Allison Flanagan, Rajeshwari Jakkam, Katherine Hansen, Kimberlee Gauvreau, Naomi Gauthier

**Affiliations:** Department of Cardiology, Boston Children’s Hospital, Boston, MA, United States

**Keywords:** mindset, exercise capacity, physical activity, congenital heart disease, meaning and purpose, exercise training

## Abstract

**Introduction:**

Patients with congenital heart disease (CHD) have variable degrees of peak oxygen consumption (VO_2_) that can be improved with supervised fitness training. The ability to exercise is affected by anatomy, hemodynamics, and motivation. Motivation is in part related to mindset, or personal attitudes and beliefs, and a more positive mindset around exercise has been associated with better outcomes. It is unknown whether variations in measured peak VO_2_ in patients with CHD are related to having a positive mindset.

**Methods:**

Patient's ages 8–17 years with CHD were administered quality of life and physical activity questionnaires at the time of their routine cardiopulmonary exercise test. Those with severe hemodynamic burden were excluded. Patients were grouped based on disease classification. Mindset was evaluated via validated questionnaires including a PROMIS Meaning and Purpose (MaP) survey and an Anxiety survey. Pearson correlation coefficients were calculated to estimate the magnitude of the association between percent predicted peak oxygen consumption (pppVO_2_) and questionnaire scores overall and within CHD subgroups.

**Results:**

Eighty-five patients participated; median age was 14.7 years, 53% were female, 66% had complex CHD, 20% had simple CHD, and 14% had single ventricle heart disease. Mean MaP scores were significantly lower in all CHD groups compared to population norms (*p* < 0.001). As a group, MaP scores were positively associated with the amount of reported physical activity (*p* = 0.017). In patients with simple CHD, MaP scores were positively associated with pppVO_2_ (*p* = 0.015). The association was even stronger for MaP:Anxiety, with worse ratios associated with lower pppVO_2_ (*p* = 0.005). Patients with complex and single ventricle CHD did not show a similar association.

**Conclusions:**

Patients with CHD, regardless of severity, had lower meaning and purpose scores than the general population, and these scores were associated with amount of reported physical activity. In the simple CHD subset, having a more positive mindset was associated with higher peak VO_2_ and a more negative mindset with lower peak VO_2_. This relationship was not seen with more significant CHD. While underlying CHD diagnoses are not modifiable, mindset and peak VO_2_ are, and consideration should be given to measuring both as each may be a target for intervention.

## 1. Introduction

Peak oxygen consumption (VO_2_) is the measurement of the ability of the cardiopulmonary system to meet the metabolic needs of the body with the increasing demands of exercise, and is measured by gas exchange analysis on a cardiopulmonary exercise test (1, 2). Peak VO_2_ is important to measure in patients with congenital heart disease as it has been shown to relate to outcomes, can assess response to treatment, and can aid in medical decision making; associations of peak oxygen consumption with quality of life have been more variable (2–7). Peak VO_2_ is an excellent marker of cardiorespiratory fitness for children with and without congenital heart disease (CHD) and is related to baseline anatomic, hemodynamic, and genetic factors. Improving cardiorespiratory fitness through exercise training requires regular and sustained attention, particularly around intensity and duration of exercise (8, 9).

Since most youth with CHD are not involved in supervised, structured exercise training (10–12), cardiorespiratory fitness level as measured by peak VO_2_ in clinical practice should reflect the summation of the patient's own “exercise training” done in their daily lives combined with the hemodynamic burden related to their congenital heart disease at the time of testing. It is important to note, physical activity is defined as “any bodily movement produced by skeletal muscles that results in energy expenditure” and exercise is a subcomponent of physical activity that is planned, structured and repetitive in nature (13). In order to affect peak oxygen consumption, exercise must be of sufficient duration, intensity and volume, which is not the same as measuring degrees of physical activity (8, 14, 15). While amount of exercise has been difficult to estimate, it is clear that most patients with CHD are not participating in recommended daily levels of physical activity (16).

Motivating patients to participate in physical activity and exercise is challenging, and increasing motivation is central to health interventions requiring behavior change (16, 17). In turn, motivation regarding physical activity and exercise relates to an individual's mindset (18). A positive mindset towards exercise is associated with increased engagement (19, 20). Mindset is defined as “lenses or frames of mind that orient an individual to a particular set of associations and expectations” (21). Mindsets are influenced by personal, cultural, social, and situational inputs, and are malleable and can change over time (22). Harnessing the power of mindsets or beliefs to drive outcomes has been the center of research and interventions in numerous fields such as growth mindset for education, self-efficacy and grit for athletes and executives, optimistic mindset for psychology, and placebo effect for medicine. For instance, teachers given a positive expectancy mindset that certain students would shine based on fake aptitude scores unwittingly saw their students actually gain greater IQ scores over the course of the school year despite being no different than their peers (23). In one study of mindsets regarding food, participants told they were drinking an “indulgent” higher calorie milkshake had greater satiety measured by a steep decline of the gut hormone ghrelin vs. those told it was a “sensible” low calorie milkshake, even though both groups received identical milkshakes (24). In another study regarding activity adequacy mindsets, the effect of surreptitiously displaying lower than actual step counts on a fitness tracker found that those with deflated “inadequate” step counts showed higher blood pressure and heart rate, poorer dietary habits, and lower affect and self-esteem than those who saw unaltered accurate “adequate” step counts (25). On the upside, research in psychology literature in athletes and non-athletes alike has demonstrated that an optimistic mindset is associated with better cardiorespiratory fitness, enhanced training results, and increased resilience to stress (26–31).

In adult literature, numerous health benefits have been shown to be associated with aspects of positive mindsets that lead to positive psychological well-being such as here-and-now “hedonic” markers (like happiness, satisfaction, and positive emotions) as well as future facing “eudaimonic” measures (including greater sense of meaning, growth, and purpose) (32–35). Specifically, simply having a greater sense of meaning and purpose in life is associated with lower all-cause mortality, and improvements in meaning and purpose scores relate to improved health behaviors over time (36, 37). Similarly, “ikigai,” a Japanese term referring to a sense of positive purpose or “reason to get up in the morning” has been associated with lower risk for morbidity and mortality and may play a mediating role in motivation and health behavior changes, such as increasing physical activity and exercise (36, 38). There have been attempts to measure physical and psychosocial elements of quality of life in children and adolescents with congenital heart disease using standard, validated health-related and functional status quality of life instruments including the Pediatric Quality of Life Inventory (PedsQL), Pediatric Cardiac Quality of Life Inventory (PCQLI), KINDL®, 36-Item Short Form Health Survey (SF-36), surveys, Child Health Questionnaire™ (CHQ), Child Behavior Checklist, Congenital Heart Adolescent and Teenage (CHAT) questionnaire, and ConQL surveys (6, 39). However, none of these instruments ask prospective, future facing “eudaimonic” questions to assess sense of purpose, hope, or optimism; rather, they relate to reflections regarding status at the time the survey (“here-and-now”) or to retrospective recall one week, four weeks, six months, or up to one year before the survey date.

To our knowledge, the degree to which simply looking forward with a positive mindset impacts cardiorespiratory fitness in patients with congenital heart disease is not known. For the present study, we sought to explore the potential relationship between mindset and physiological outcomes by investigating the correlation between sense of purpose and percent predicted peak oxygen consumption as measured on a standard cardiopulmonary exercise test in patients with CHD. The term “positive mindset” can be variably defined. For the purposes of this study, it was defined as beliefs regarding future-facing hopes and dreams and assessed utilizing a validated pediatric Meaning and Purpose scale. We hypothesized that as long as the patients did not have significant hemodynamic limitations, greater positive mindset would be associated with higher peak VO_2_. Investigating this association is important as mindset is modifiable and can be a potential target for an intervention to improve health in children with CHD.

## 2. Methods

This is a cross-sectional study to explore the association between mindset and reported physical activity level, and mindset and peak oxygen consumption in patients with CHD. Eligible patients were 8–17 years of age, were primarily English speaking, had congenital heart disease, and presented for a clinically indicated cardiopulmonary exercise test (CPET). Charts were reviewed for diagnoses, interventions, recent (<1 year) clinical status, imaging data, severity of CHD, baseline hemodynamics, and other clinical data including age, gender, weight, height, chromosome abnormalities or syndromes, and noncardiac anatomic defects. Questionnaire data and exercise test data were compared to clinical data in and between disease severity groups. The study was approved by the Boston Children's Hospital Institutional Review Board.

### 2.1. Exercise testing

CPETs were performed either on the cycle ergometer using a ramp protocol or treadmill using the Bruce protocol and expired gases were measured using an Ultima CPX^TM^ metabolic stress testing system (MGC Diagnostics, St Paul, MN). Peak oxygen consumption (VO_2_) was determined by the highest reliable value obtained during exercise, and percent predicted peak oxygen consumption was determined via prediction equations adjusted for modality using standard clinical practices (2). Patients were excluded if they had non-CHD factors that could affect exercise: use of beta blockers or negative inotropes, had pacemakers or ICDs, active arrhythmias, significant musculoskeletal or pulmonary disease, or did not complete a maximal CPET defined as a respiratory exchange ratio >1.09. Patients were also excluded if they were unable to complete the questionnaires due to cognitive delays or language barrier, or had severe CHD burden that would potentially affect exercise testing: those with severe ventricular dysfunction, significant cyanosis, malignant arrhythmias, severe volume or pressure load, or severe pulmonary hypertension. Patients were recruited and consented/assented on arrival for their scheduled exercise test. Baseline questionnaires (see below) were administered during the visit for their exercise test.

### 2.2. Questionnaires

The Patient-Reported Outcomes Measurement Information System (PROMIS) surveys, developed under the auspices of the National Institutes of Health, has a “eudaimonic” Meaning and Purpose (MaP) questionnaire that has been validated in a general population of US children ages 8–17 years and was chosen as the primary variable (40). The questionnaire asks eight questions about whether patients feel hopeful, can reach goals, have a sense of purpose in life, and feel positive about their future. Further, since the general concept of resilience in children may be the summation of positive and negative inputs (31) which together may contribute to mindsets toward physical activity, a negative factor (the Pediatric Anxiety scale, eight questions that assess the degree of state anxiety felt in the past week) was also assessed. Two additional “hedonic” PROMIS questionnaires were chosen: Life Satisfaction as a marker of current status and Positive Affect to assess recent past positive functioning and friendships (41, 42). The study also assessed self-reported Physical Activity (43). Raw scores for each instrument were translated to t-scores with mean 50 and standard deviation 10 (44, 45). Finally, a standard quality of life tool for congenital heart disease, the Pediatric Cardiac Quality of Life Inventory (PCQLI), was used to compare our study population with populations previously evaluated in the literature (46). The advantage of the PCQLI is that it has been validated and used for CHD. The disadvantage, and reason it could not be used for this study as the primary variable, is that it asks retrospective questions and is oriented toward identifying negative thoughts or concerns, whereas the PROMIS Meaning and Purpose (MaP) scale assesses positive mindsets about the future.

### 2.3. CHD severity groups

While paradigms exist for standardized risk categorization of patients who have had cardiac surgery, there is no such uniformly accepted categorization for patients with CHD more broadly (those with or without having had surgery). Most studies devise systems based on subjective “trivial/mild” “moderate” and “severe” categories, often also separating out patients with a functional single ventricle into their own category (47–49). For the purposes of our study, we adopted the classification set forth by Qu et al. (49) in their study of CPET parameters across a range of CHD complexities in which they divided patients into three anatomic subgroups: simple congenital heart disease (SCHD), complex congenital heart disease (CCHD) and single ventricle heart disease (SVHD). See Supplementary Material for disease classification breakdown among our study cohort. For secondary analysis, each subgroup was further categorized by hemodynamic rather than anatomic classification in which function, oxygen levels, rhythm, complex anatomy, and elevated load (FORCE) were assessed to determine hemodynamic burden (50). Patients were stratified as FORCE 1 or FORCE 2. By design, patients who met FORCE 3 criteria, those with the most severe hemodynamic burden, were excluded.

### 2.4. Analysis

The PROMIS variables included: Pediatric Meaning and Purpose (MaP) Scores, Pediatric Anxiety Scores, and the ratio of Meaning and Purpose Score to Anxiety Score. PROMIS surveys are scored such that the higher the number, the more of the trait in question. Higher purpose is represented by a high score on the MaP scale and lower anxiety is represented by a lower score on the Anxiety scale. For our study, participants with high MaP:Anxiety ratios (greater than 1) were labeled as having “more positive” mindsets, while those with low ratios (less than 1) were labeled as having “less positive” mindsets. The primary clinical variable measured was percent predicted peak oxygen consumption (pppVO_2_).

Other variables of interest included PROMIS Pediatric Life Satisfaction Scores*,* PROMIS Pediatric Positive Well Being Scores and the PCQLI Questionnaire scores. We also explored the role of physical activity, as assessed by the PROMIS Physical Activity Questionnaire, in the relationship between mindset and pppVO_2_. Other related variables of interest were submaximal exercise values: heart rate at ventilatory anaerobic threshold (VAT), VO_2_ at VAT, V_E_/VCO_2_ slope, end tidal CO_2_ and PROMIS Physical Activity questionnaire scores.

Although our aims are exploratory, we wanted to have sufficient power to detect a modest correlation in the cohort as a whole, if such an association existed. A sample of size 85 provides 80% power to detect a correlation of 0.30 or higher using a two-sided test conducted at the 0.05 level of significance.

Categorical variables were summarized using frequencies and percentages, and continuous variables were summarized using the median and either the range or interquartile range, or the mean and standard deviation as noted. Patient characteristics and exercise data are compared across CHD subgroups using Fisher's exact test or the Kruskal-Wallis test, and PROMIS scores using one-way analysis of variance. Comparisons of mean PROMIS and PCQLI scores to population norms were made using the one-sample *t*-test. For the ratio of meaning and purpose to pediatric anxiety, the comparison was made to a mean value of 1, which would be the value assumed if the two scores were identical. Pearson correlation coefficients were calculated to estimate the association between pppVO_2_ and PROMIS scores overall and within CHD subgroups. Linear regression was used to adjust for baseline patient characteristics when appropriate.

### 2.5. Adjustment for the COVID-19 pandemic

It is important to note this study started prior to the COVID-19 pandemic which may have had a negative impact on quality of life or mindset, both of which are measured in this study. An amendment to the protocol was made that allowed study personnel to re-consent and re-administer the surveys to those who participated prior to March 2020. Pre- and Post-COVID survey data were compared using the paired *t*-test to look for differences in PROMIS and PCQLI survey data.

## 3. Results

### 3.1. Participant characteristics

In total, 90 participants were consented. One patient was excluded for not reaching the maximal exercise test criteria (RER > 1.09) and four patients were deemed ineligible after consent due to final results of echocardiographic data done the same day as the CPET reclassifying the patient into a more severe CHD classification (FORCE 3) that excluded them from participating, yielding a total of 85 patients for analysis. The median age was 14.7 years, 53% were female, 20% had SCHD, 66% CCHD and 14% SVHD. Other participant characteristics are reported in Table 1. Other than height (*p* = 0.033), there were no significant differences in baseline characteristics between SCHD, CCHD, and SVHD.

**Table 1:** Descriptive statistics of participant characteristics (*n* = 85).

Age at study (years)	14.7 (8.3, 17.8)
**Age group**	
Pre-teen	22 (26%)
Teen	63 (74%)
**Sex**	
Male	40 (47%)
Female	45 (53%)
Weight (kg)	51.2 (29.8, 120.4)
Height (cm)	161 (127, 187)
Chromosomal abnormality	4 (5%)
Any non-cardiac abnormality	31 (36%)
**CHD severity**	
Simple CHD	17 (20%)
Complex CHD	56 (66%)
Single Ventricle Heart Disease	12 (14%)

Values shown are in number (percent) or median (range).

### 3.2. Patient characteristics pre- and post-COVID-19 pandemic outbreak

There were 14 patients who participated in the study prior to the onset of the global COVID-19 pandemic. Due to the nature of the surveys we attempted to re-consent and re-administer the surveys for the 14 patients who participated prior to March 2020 to compare to those who solely participated post-pandemic onset. We were able to reach and re-consent eight patients to complete a second set of surveys. The median age of these patients was 14.7 years; 75% were female, 12% simple CHD, 25% complex CHD and 63% single ventricle CHD. There were no significant differences in mean MaP (*p* = 0.46), pediatric anxiety (*p* = 0.41), MaP to anxiety ratio (*p* = 0.99), positive affect (*p* = 0.22) and physical activity (*p* = 0.60) from pre- to post-COVID-19 pandemic. There was, however, a significantly lower life satisfaction score (*p* = 0.033) post-pandemic. We thus excluded all life satisfaction data in the final analysis.

To evaluate whether our cohort was similar to or different than CHD populations reported in the literature, we compared our cohort to a standard quality of life tool (PCQLI). We were unable to analyze pre- and post-COVID-19 pandemic PCQLI scores due to missing data. While we were unable to compare pre-pandemic PCQLI scores to post-pandemic ones, unlike life satisfaction, we found no significant difference in our post-pandemic PCQLI scores to the general population reported in the literature prior to the global pandemic (Table 2) and thus chose to report post-pandemic values (pre-teen *n* = 17, teen *n* = 53; one post-pandemic participant had missing PCQLI data).

**Table 2:** PROMIS and PCQLI scores compared to population norms within all CHD patients.

PROMIS (*n* = 85)	CHD cohort	Reference value	*p*-value
Meaning and Purpose	46.4 ± 7.3	50	<0.001
Pediatric Anxiety	50.6 ± 8.5	50	0.74
MaP: Anxiety Ratio	0.95 ± 0.28	1	0.067
Positive Affect	48.8 ± 7.3	50	0.066
Physical Activity (*n* = 84)	48.6 ± 8.4	50	0.061
**PCQLI: Pre-Teen (*n* = 17)**			
PCQLI Total Score	70.6 ± 16.5	73.83	0.44
PCQLI Disease Impact Subscale Score	34 ± 7.7	36.90	0.14
PCQLI Psychosocial Impact Subscale Score	36.6 ± 9.2	36.92	0.89
**PCQLI: Teen (*n* = 53)**			
PCQLI Total Score	77.9 ± 15.6	79.68	0.41
PCQLI Disease Impact Subscale Score	38.4 ± 7.8	38.43	0.98
PCQLI Psychosocial Impact Subscale Score (*n* = 53)	39.6 ± 8.6	41.26	0.17

### 3.3. Cardiopulmonary exercise test data

The overall median pppVO2 was 80%, heart rate at ventilatory anaerobic threshold (VAT) was 125 bpm, VO_2_ at VAT 17.6 ml/kg/min, VE/VCO_2_ slope 28 and PETCO_2_ at VAT 38. Median pppVO_2_ differed significantly among the diagnosis groups (*p* = 0.01); it was lower for subjects with a diagnosis of single ventricle (72%) than for either simple or complex CHD (79% and 83%, respectively). Median heart rate at VAT also differed among groups (*p* = 0.02); it was lowest for single ventricle patients (119 bpm) and higher for simple and complex CHD (131 bpm and 128 bpm). Median VO_2_ at VAT also differed among groups (*p* = 0.008), it was lowest for single ventricle patients (16.6 ml/kg/min) and higher for simple and complex (20.7 ml/kg/min and 17.5 ml/kg/min). Median VE/VCO_2_ slope also differed among groups (*p* = <0.001), it was highest for single ventricle patients (34) and lower for simple and complex (27 and 27), and lastly median PETCO_2_ at VAT also differed among groups (*p* < 0.001), it was lowest for single ventricle patients (32) and higher for simple and complex (40 and 38).

### 3.4. Meaning and purpose and quality of life scores

The means and standard deviations for study participants self-reported PROMIS survey data and for PCQLI by age group are reported in Table 2. The mean MaP score is significantly lower than that of the general population (*p* < 0.001). The ratio of meaning and purpose to pediatric anxiety is lower than that of the general population (assuming a mean of 1 in the general population) but does not achieve statistical significance. Mean scores for positive affect and physical activity are also lower, but do not achieve statistical significance. The mean pediatric anxiety score, PCQLI total scores and PCQLI subscale scores were not significantly different from the reference population.

There were no statistically significant differences noted between CHD subgroups for meaning and purpose (*p* = 0.10); anxiety (*p* = 0.90); MaP:Anxiety ratio (*p* = 0.51); positive affect (*p* = 0.36); physical activity (*p* = 0.17); Pre-Teen PCQLI total score (*p* = 0.47); Pre-Teen PCQLI disease impact subscale score (*p* = 0.35); Pre-Teen PCQLI psychosocial impact score (*p* = 0.59); Teen PCQLI total score (*p* = 0.17); Teen PCQLI disease impact subscale score (*p* = 0.07); or Teen PCQLI psychosocial impact score (*p* = 0.39).

When the CHD subgroups were compared to the general population norms, MaP was significantly lower across all subgroups, including simple CHD (*p* = 0.003), complex CHD (*p* = 0.004) and single ventricle CHD (*p* = 0.021, Table 3). In those with simple CHD, the ratio of meaning and purpose to pediatric anxiety (*p* = 0.030) and positive affect (*p* = 0.030) were also significantly lower than the population norm. Simple CHD trended towards lower physical activity than the general population (*p* = 0.053), which was not the case for those with complex and single ventricle CHD. In addition, anxiety scores were not significantly different than the general population for each subgroup (Table 3).

**Table 3:** PROMIS scores compared to population norms within each CHD subgroup.

	SCHD (*n* = 17)	*p*-value	CCHD (*n* = 56)	*p-*value	SVHD (*n* = 12)	*p*-value
Meaning and Purpose	44.5 ± 7.3	0.003	47.6 ± 6.5	0.004	43.5 ± 9.8	0.021
Pediatric Anxiety	50.5 ± 5.6	0.65	50.8 ± 9.1	0.75	49.6 ± 9.6	0.44
MaP:Anxiety Ratio	0.90 ± 0.21	0.030	0.98 ± 0.29	0.29	0.92 ± 0.32	0.21
Positive Affect	47.3 ± 5.5	0.030	49.6 ± 7.5	0.35	47.1 ± 8.6	0.13
Physical Activity	45.3 ± 11.2	0.053	49.7 ± 7.7	0.38	48.0 ± 6.0	0.13

### 3.5. Relationship of meaning and purpose and quality of life to physical activity outcomes

For the entire cohort, there was a positive association between MaP and reported physical activity (*r* = 0.26; unadjusted regression coefficient 0.30; *p* = 0.017). This remained statistically significant after adjusting for age, sex, CHD type, and FORCE (adjusted coefficient 0.27; *p* = 0.037, Table 4). In the multivariable model, none of the other patient characteristics were significantly associated with reported physical activity. No other statistically significant associations with reported physical activity were seen, including for pediatric anxiety, MaP to anxiety ratio, or positive affect. In addition, no association was noted between reported physical activity and other PROMIS scores within each group.

**Table 4:** Relationship between reported physical activity and meaning and purpose.

Outcome: Reported physical activity	Univariate linear regression models			Multivariable linear regression model		
	Coefficient	95% confidence interval	*p*-value	Coefficient	95% confidence interval	*p*-value
Meaning and Purpose	0.30	0.06, 0.54	0.017	0.27	0.02, 0.52	0.037
Age at study ↑1 year	−0.19	−0.96, 0.57	0.61	−0.24	−1.00, 0.51	0.52
Sex female	−0.75	−4.44, 2.93	0.69	−1.37	−5.02, 2.28	0.46
**CHD (vs. simple)**						
Complex	4.36	−0.25, 8.98	0.064	3.74	−0.93, 8.42	0.11
Single ventricle	2.65	−3.63, 8.92	0.40	3.26	−3.18, 9.70	0.32
FORCE 2	−0.99	−4.67, 2.70	0.60	−0.92	−4.67, 2.84	0.63

### 3.6. Relationship of meaning and purpose and quality of life to percent predicted peak oxygen consumption

**Figure 1:**
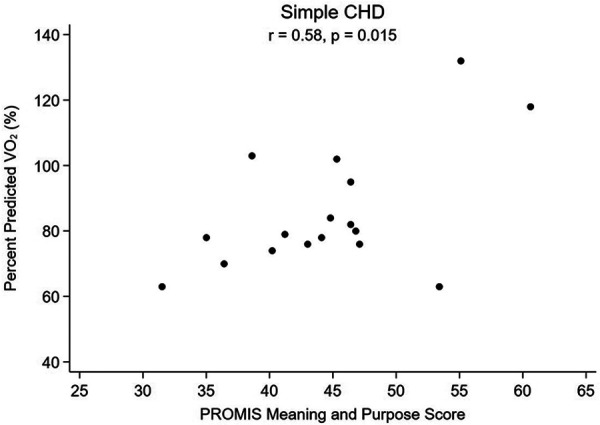
Percent predicted VO2 vs. Meaning and Purpose, Simple CHD.

**Figure 2:**
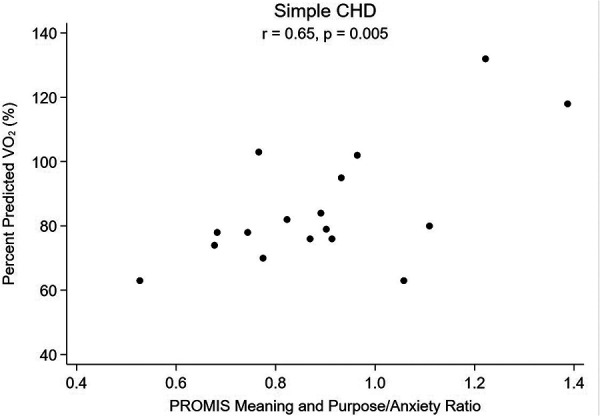
Percent predicted VO2 vs. Meaning and Purpose to Anxiety Ratio, Simple CHD.

For patients with simple CHD, an association was seen between higher pppVO2 and greater MaP (*r* = 0.58; *p* = 0.015, Figure 1), less anxiety (*r* = −0.49; *p* = 0.048) and improved MaP to anxiety ratio (*r *= 0.65; *p* = 0.005, Figure 2). When adjusting simple CHD for hemodynamic burden (FORCE category), meaning and purpose remains significant (adjusted coefficient 1.70; *p* = 0.010, Table 5). For patients with greater CHD anatomic burden (single ventricle heart disease and complex CHD), a similar association was not seen. Positive affect and physical activity was also not associated with pppVO_2_ for any CHD subgroup (Table 6).

**Table 5:** Relationship between percent predicted peak VO_2_ and meaning and purpose for patients with simple CHD.

Outcome: percent predicted peak VO_2_	Univariate linear regression models			Multivariable linear regression model		
	Coefficient	95% confidence interval	*p*-value	Coefficient	95% confidence interval	*p*-value
Meaning and Purpose	1.48	0.33, 2.63	0.015	1.70	0.49, 2.92	0.010
FORCE 2	−1.04	−21.4, 19.3	0.91	−9.46	−27.1, 8.15	0.27

**Table 6:** Pearson correlation coefficients with percent predicted peak VO_2_ (*p*-value).

PROMIS (*n *= 85)	Percent predicted peak VO_2_			
	Total (*n* = 85)	SCHD (*n* = 17)	CCHD (*n* = 56)	SVHD (*n* = 12)
Meaning and Purpose	0.19 (0.074)	0.58 (0.015)	0.04 (0.76)	0.05 (0.88)
Pediatric Anxiety	−0.05 (0.65)	−0.49 (0.048)	−0.04 (0.80)	0.42 (0.17)
MaP:Anxiety Ratio	0.13 (0.24)	0.65 (0.005)	0.05 (0.74)	−0.24 (0.46)
Positive Affect	0.03 (0.76)	0.27 (0.29)	−0.04 (0.79)	−0.10 (0.76)
Physical Activity	0.19 (0.088)	0.19 (0.47)	0.19 (0.16)	0.50 (0.10)

**Figure 3:**
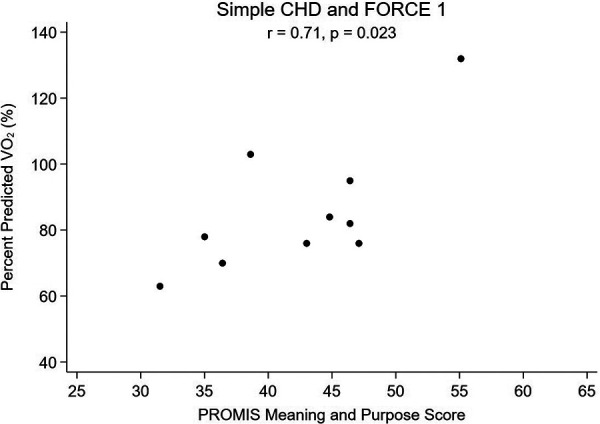
Percent predicted VO_2_ vs. Meaning and Purpose, Simple CHD and FORCE 1.

**Figure 4:**
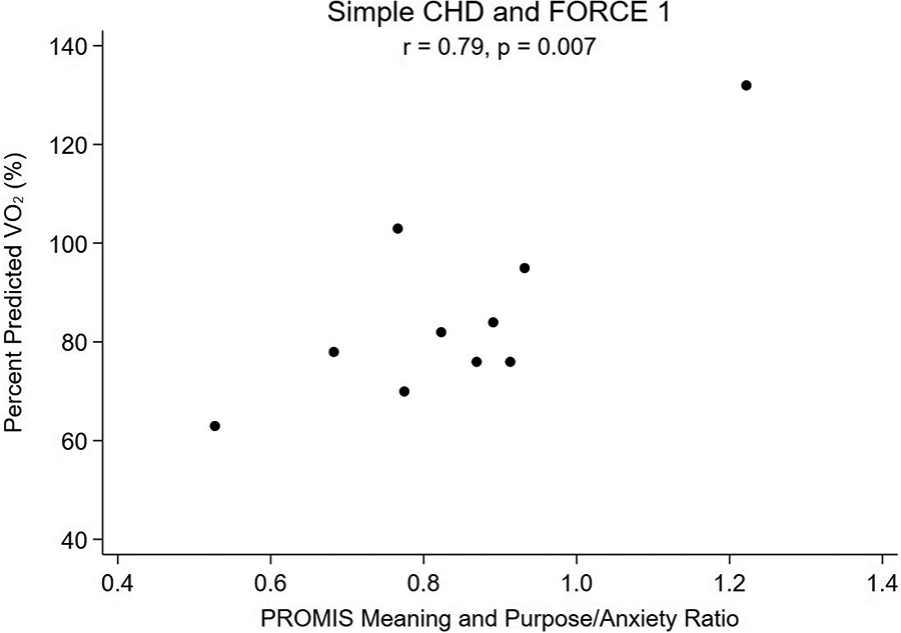
Percent predicted VO_2_ vs. Ratio of Meaning and Purpose to Anxiety, Simple CHD and FORCE 1.

Lastly, a subanalysis subdividing SCHD and CCHD into FORCE 1 and FORCE 2 categories to explore the relationship between meaning and purpose score and pppVO_2_ was performed. The subanalysis was not performed for SVHD patients as the sample size was too small. Patients with SCHD who were FORCE 1 and had greater MaP (*r* = 0.71; *p* = 0.023, Figure 3) and more favorable MaP to anxiety ratio (*r* = 0.79; *p* = 0.007, Figure 4) also had a higher pppVO_2_. Patients with greater hemodynamic burden (SCHD with FORCE 2 and CCHD of either FORCE category) did not show a similar association, as seen in Table 7.

**Table 7:** Pearson correlation coefficients with percent predicted peak VO_2_ (*p*-value).

PROMIS (*n* = 85)	Percent Predicted Peak VO_2_			
	SCHD + FORCE 1 (*n* = 10)	SCHD + FORCE 2 (*n* = 7)	CCHD + FORCE 1 (*n* = 32)	CCHD + FORCE 2 (*n* = 24)
Meaning and Purpose	0.71 (0.023)	0.50 (0.25)	0.24 (0.19)	−0.12 (0.58)
Pediatric Anxiety	−0.55 (0.10)	−0.47 (0.29)	−0.15 (0.41)	0.06 (0.78)
MaP:Anxiety Ratio	0.79 (0.007)	0.61 (0.15)	0.18 (0.32)	−0.04 (0.84)
Positive Affect	0.35 (0.33)	0.23 (0.61)	0.10 (0.59)	−0.23 (0.28)

## 4. Discussion

While there is a growing body of literature in adult heart disease on the impact of overall positive mindset and psychological well-being, including specifically sense of purpose in life, being related to better physical activity and lower cardiovascular risk (34, 35, 51), there is very little research exploring the potential association between mindset and cardiac health in pediatric patients with CHD. We hypothesized that patients with CHD without hemodynamic burden that would limit exercise and who had a positive mindset may be more motivated and more likely to participate in greater volume or intensity of exercise in their daily lives, and thus may have a higher measured peak oxygen consumption as a result. Similarly, we hypothesized that those with lower mindset scores may have lower baseline percent predicted peak VO_2_. In other words, we postulated that “those who think they can, will, and those who think they ca’t, won’t” regarding engaging in purposeful exercise and developing better cardiorespiratory fitness. Indeed, we found an association between higher sense of purpose and percent predicted peak VO_2_, with a more positive mindset associated with a more positive physical measure of fitness (more positive peak VO_2_) and a more negative mindset associated with a more negative peak VO_2_, as long as there is milder CHD (Figure 1). The potential association between mindset and peak VO_2_ had a higher correlation coefficient when we further subdivided patients into both the lowest anatomic and lowest hemodynamic burden (Figure 3). Interestingly, for patients with more advanced CHD (those with complex CHD or single ventricle heart disease), the effect of meaning and purpose on pppVO_2_ was attenuated; no association was found. While our study did not seek to assess mechanisms, we postulated that there could be a point in which hemodynamic limitations that could affect peak exercise in patients with CCHD and SVHD cannot be overcome by mindset alone. For those patients, supervised, directed exercise training may be required as it has been shown that these programs can improve peak VO_2_ more than individuals can achieve on their own across a range of severities of congenital heart disease (10, 12, 52, 53) and those effects can be sustained (54, 55).

Additionally, we were interested in the balance of factors within each individual, and if the factors measured in the pediatric meaning and purpose scale such as having hopes, positive feelings about the future, and a feeling that goals are in reach would be tempered by co-existing levels of state anxiety. It is important to note that mindset, or beliefs, are distinct from both mental health (a state of being) and mental illness/mental health disorders (diagnosable conditions). Literature on resiliency and looking through a trauma informed lens has suggested that a more resilient mindset can lessen effects of mental health disorders such as depression; for example, a study on Syrian refugee children and adolescents suggested that having a resilient mindset was actually a strong protective factor against symptoms of depression (56) and encouraging steps to enhance resiliency could be a target for intervention for youth (31). Children with CHD are known to have significantly higher risk of mental health disorders including trait anxiety than the general population (57), and we hypothesized that the ratio of positive mindset to state anxiety may be important as it relates to cardiorespiratory fitness. We did not find an increase in baseline self-reported state anxiety for our cohort, but we did find that patients with more favorable meaning and purpose to anxiety ratios had the highest correlations with better peak VO_2_, especially in patients with little to no anatomic and hemodynamic limitations (Figures 2, 4). This warrants further study as mindset is modifiable so if it can be improved, and anxious feelings lessened, then perhaps there could be a direct health outcome (percent predicted peak VO_2_) that could be improved as well. Systematically measuring mindset in our CHD population as part of “quality of life” tools would allow more opportunities to study such associations.

The relationship between well-being and health is complex, as are the concepts of motivation, self-efficacy, and responses to achieving goals (33), which are of particular importance as they relate to engaging in physical activity at intensity levels necessary to improve cardiorespiratory fitness. Specifically, having a strong sense of purpose relates to being physically active, and sense of purpose and physical activity positively increase the effect of one on the other. In one longitudinal cross-lagged panel model of over 14,000 adults looking at the relationship between sense of purpose and physical activity, Yemiscigil and Vlaev found that people with greater sense of purpose in life were more physically active, and the added physical activity yielded a greater sense of purpose as the two amplified the effects of one other in a bi-directional fashion over time (58). For our cohort, we found greater reported physical activity was associated with better meaning and purpose scores. This was not found when we did this analysis within CHD subgroups. Whether this was due to limitations in power in our study or due to the nature of reported (rather than measured) physical activity is unclear. In one study of 343 children with simple, moderate, and complex CHD in which physical activity was quantified by wearable fitness trackers, greater physical activity was positively associated with positive well-being (59). In a meta-analysis of studies utilizing a physical activity intervention in healthy children, physical activity was also associated with enhancing participant self-esteem (60). In contrast, another systematic review of 33 articles on cardiorespiratory exercise capacity, physical activity, and quality of life measures including psychosocial domains specifically in patients with CHD, results were mixed and no clear associations were found (6). The quality of life measures used in these studies did not include forward facing meaning and purpose scales however, which may influence their results.

Associations between severity of CHD, self-efficacy, and physical activity have been investigated previously. In one study of 100 adolescents in Israel with trivial, mild, or moderate CHD, it was self-efficacy and not severity of disease that was associated with participation in physical activity and sport (61). Another study out of Canada evaluated 137 patients with congenital heart disease of either simple (atrial septal defects) or more complex (transposition of the great arteries and tetralogy of Fallot) or functionally single ventricle with Fontan palliation subtypes. They found that higher moderate to vigorous physical activity (MVPA) levels as measured by accelerometers were associated with higher percent predicted peak VO_2_ and greater self-efficacy across all ranges of CHD subtypes (62). In a previous study done by the same group in patients with a Fontan circulation, they found that peak VO_2_ (exercise capacity) was more associated with measures of general health and self-esteem than minutes of MVPA (15). With this in mind, they felt that “while peak exercise capacity may be associated with MVPA, other factors may be more important in predicting peak VO_2_ than MVPA alone” (62). Whether having a greater sense of meaning and purpose may comprise a portion of the missing association is not yet known but is interesting to consider. We did demonstrate that patients with CHD have lower meaning and purpose scores compared to the general population. Across all levels of disease severity (SCHD, CCHD, SVHD), patients in our cohort rate themselves worse than other US children in terms of meaning and purpose, and those with simple CHD disease also displayed a lower meaning and purpose to anxiety ratio and less positive affect. Our cohort appears to be similar to other CHD patients evaluated in the literature in terms of quality of life as measured by PCQLI scores (63). We found that patients with “simple” CHD and the least hemodynamic burden with the lowest sense of purpose had the lowest peak VO_2_, and those with the highest sense of purpose had the highest peak VO_2_. Thus patients who are considered “low risk” by their providers based on their anatomy and hemodynamic burden may have low fitness levels and less positive mindsets that would be unappreciated if mindset and peak VO_2_ are not evaluated. It was notable to us that in our sample, patients with simple CHD made up a smaller fraction of patients presenting for CPETs recruited for our study. Whether this is a selection bias or reflects a trend that providers may not think to order CPETs in patients with mild disease was not evaluated in our study.

There are many physiological and musculoskeletal factors that can contribute to reduced cardiorespiratory fitness, as can psychosocial factors such as anxiety and depression, perception of disease status, disease stigmatization, and exclusion from sports and physical activity at school and within their community. Children are hard-wired biologically to play, yet children with chronic health conditions may face extrinsic and intrinsic barriers to recreational activity with the potential for far-reaching negative consequences on health and development (64). Currently, cultural and social influences have resulted in healthy children becoming distressingly sedentary; those with CHD are no better, with lower to equivalently low levels of activity as their peers without CHD (65, 66). Unfortunately, parents and health care providers feel unprepared to encourage and promote physical activity, and research and guidance remains limited (16). It is known, however, that supervised exercise training can improve peak oxygen consumption, and it is also known that mindsets can be trained or influenced for both positive and negative health behaviors (19, 20). Promoting physical activity and inspiring motivation and belief in the ability to succeed are important elements in helping patients across the range of CHD severity establish exercise habits (16, 67). Exploring interventions that improve mindset, decrease anxiety, and improve fitness all together would be important future work.

## 5. Limitations

One of the greatest limitations of our study was our small sample size which may have had an impact on some of the associations made among these groups. Another limitation of our study was that due to the nature of our study design, we only utilized self-reported physical activity which has known limitations and inaccuracies. All patients utilized the same PROMIS measures but tracking actual physical activity would have strengthened the data. Also, the COVID-19 pandemic occurred during the enrollment phase of the study which could have affected our results given the nature of survey items asked. To assess for this, we were able to re-consent eight of the 14 patients who had participated initially before the pandemic and compared their results. We found no significant differences from pre- to post-pandemic other than for Life Satisfaction. We were interested in this variable but unfortunately did not feel we had adequate data to analyze the effect of the pandemic itself and chose to exclude it. It would be interesting to further evaluate this variable in the future. Another limitation was that we did not account for timing of CHD diagnosis. We do not know if there is a difference between the mindsets of patients with a later diagnosis of CHD such as a coronary anomaly or atrial septal defect compared to those who were diagnosed at birth and never knew themselves from any other perspective. This would be an interesting aspect to study in the future. We also did not evaluate any differences in males to females or ages (pre-teen to teen) for the PROMIS measures. Finally, defining “mindset” in terms of meaning and purpose may be too narrow and restrictive considering the body of literature regarding mindsets, as sense of purpose only partially informs beliefs. A limitation in our study was that no prior literature has defined “positive mindset” in terms of meaning and purpose scores for the CHD population.

## 6. Conclusion

Patients with CHD have lower meaning and purpose scores than the general population. These scores are associated with peak VO_2_ in patients with simple CHD and little to no hemodynamic burden, with higher scores associated with better percent predicted peak VO_2_ and lower scores associated with lower percent predicted peak VO_2_. The balance of positive mindset to anxiety is also associated with peak VO_2_. Whether that means these children with a positive mindset approach life with more gusto, and thus are more likely to exercise more or at higher intensities or avail themselves of opportunities for fitness, was not evaluated in our study but warrants further research. The relationship of the forward facing “meaning and purpose” positive mindset variable to measured peak VO_2_ in pediatric patients with CHD has not been previously explored in the literature to our knowledge, and systematically assessing mindset and peak VO_2_ as part of health related quality of life assessment may provide new insights and potential targets for treatment.

## Data Availability

The original contributions presented in the study are included in the article/**Supplementary Material**, further inquiries can be directed to the corresponding author.
