# Supplementary material for: Positive mindset and exercise capacity in school-aged children and adolescents with congenital heart disease
Source: Front Pediatr. 2023 Jun 2;11:1133255. doi: 10.3389/fped.2023.1133255 (PMC10272603; doi:10.3389/fped.2023.1133255)
Supplement: Supplementary file 1 [file Datasheet1.pdf]

Table S1. Positive Mindset and Exercise Capacity Disease Classification Breakdown

Classification	N = 85	Original Diagnosis
Simple	17	Atrial septal defect, anomalous origin of the right coronary artery
		Mitral valve prolapse
		Subaortic stenosis (n=2)
		Atrial septal defect
		Aortic stenosis
		Mitral regurgitation
		Bilateral branch pulmonary artery stenoses
		Atrial septal defect, mild left ventricular noncompaction
		Bicommissural aortic valve, mild aortic regurgitation, small patent ductus arteriosus
		Cleft mitral valve
		Aortic stenosis and regurgitation
		Atrial septal defect, ventricular septal defect, coarctation of the aorta
		Pulmonary stenosis (n=2)
		Bicommissural aortic valve
		Unicommissural aortic valve
Complex	56	Left pulmonary vein atresia, hypoplastic left pulmonary artery, hypoplastic left lung
		Total anomalous pulmonary venous connection
		Double-chambered right ventricle, ventricular septal defect, subpulmonic stenosis
		Tetralogy of Fallot (n=12)
		Tetralogy of Fallot with pulmonary atresia
		Tetralogy of Fallot with pulmonary atresia and major aortopulmonary collaterals
		Tetralogy of Fallot, pulmonary regurgitation
		Tetralogy of Fallot with absent pulmonary valve, supravalvar pulmonic stenosis, free pulmonary regurgitation
		Tetralogy of Fallot with pulmonary atresia and major aortopulmonary collaterals (n=2)
		Dysplastic pulmonary valve, pulmonic stenosis, severe pulmonary artery dilation with airway compression, atrial septal defect
		Pulmonary atresia with intact ventricular septum (n=2)
		Pulmonary atresia with intact ventricular septum, dysplastic tricuspid valve, tricuspid regurgitation
		D-transposition of the great arteries (n=6)
		D-transposition of the great arteries, aortic regurgitation
		D-transposition of the great arteries, coarctation of the aorta
		Double outlet right ventricle
		Double outlet right ventricle, ventricular septal defect, coarctation of the aorta
		Double outlet right ventricle, ventricular septal defect, subpulmonary stenosis
		Double-chambered right ventricle, ventricular septal defect
		Ebstein's anomaly
		Ebstein's anomaly, coarctation of the aorta, bicommissural aortic valve
		Ebstein's anomaly, severe tricuspid insufficiency
		Ebstein's anomaly of the tricuspid valve, secundum ASD
		Mixed aortic valve disease, coarctation of the aorta, bicommissural aortic valve
		Transposition of the great arteries, coarctation of the aorta, single coronary artery, left pulmonary artery stenosis
		Mitral stenosis, dysplastic mitral and tricuspid valve

		Dextrocardia, coarctation of the aorta, bicommissural aortic valve with aortic stenosis, mitral stenosis
		Dextrocardia, L-transposition of the great arteries with pulmonary atresia, ventricular septal defect, Mustard-Rastelli repair
		Heterotaxy syndrome, dextrocardia, transposition of the great arteries with pulmonary atresia, complete AV canal
		Critical aortic valve stenosis, Ozaki repair
		Coarctation of the aorta, ventricular septal defect
		Coarctation of the aorta, parachute mitral valve
		Atrioventricular canal type ventricular septal defect, mitral regurgitation
		Truncus arteriosus (n=2)
		Critical pulmonary stenosis, tricuspid stenosis, coronary artery fistula
		Complete AV canal
		Pulmonary atresia with intact ventricular septum
Single	12	SLL-transposition of the great arteries, tricuspid atresia, single left ventricle with sub-aortic outflow, large non-restrictive bulb ventricular foramen, and valvar pulmonary stenosis, moderate to large non-restrictive secundum atrial septal defect
		Hypoplastic left heart syndrome (n=3)
		Hypoplastic left heart variant with mitral atresia and large muscular ventricular septal defect, bicommissural aortic valve, aortic isthmus arch hypoplasia, left aortic arch with aberrant right subclavian artery, and anomalous origin of the left coronary artery from the right pulmonary artery
		L-transposition of the great arteries, valvar and supravalvar PS, tricuspid valve atresia, single left ventricle
		Heterotaxy, hypoplastic left heart syndrome with aortic atresia and mitral atresia
		Heterotaxy, unbalanced complete AV canal, double outlet right ventricle with pulmonary atresia
		Double outlet right ventricle with mitral atresia, over riding tricuspid valve, pulmonary stenosis, hypoplastic left ventricle, ASD, and AV Canal-type VSD, S/P Fontan
		Double inlet left ventricle
		Double inlet, double outlet right ventricle
		L-transposition of the great arteries, tricuspid atresia, hypoplastic right ventricle, severe pulmonary stenosis
